# Middlesex Hospital Medical School

**Published:** 1915-12-11

**Authors:** 


### PATRIOTISM IN BRITISH HOSPITALS.*

### Middlesex Hospital Medical School.

No effort has so far been made at this hospital to tabulate the names of all past students who are taking part in the war. But it is possible to study the effects of the crisis on this comparatively small medical school, and to note what has been accomplished and surrendered within the establishment for the cause.

Up to July last thirty-eight members of the staff had taken up duties in connection with the war. The process of readjustment was a gradual one, and much splendid service for the benefit of the wounded has accompanied it. As soon as war was declared the whole of the convalescent home at Clacton, together with eighty beds in the hospital, were placed at the disposal of the War Office. The number of beds at Clacton was promptly raised from ninety to 130, and the first batch of wounded were received there on September 13, 1914. Up to October last the number treated was over 2,000, and the War Office is now loudly crying out for a further increase in the number of beds. Unlike many hospitals, it was decided not to place the wounded soldiers by themselves in distinct wards at the Middlesex, but to distribute them about the hospital, and this plan has been very successful and popular with the men. Certain relaxations in the rules have been made, smoking in the wards has been allowed, and discipline does not appear to have suffered. The number of military patients treated since the outbreak of war is 355.

### Departures for the Front.

Among the first to leave the hospital was the Chairman, Prince Alexander of Teck, G.C.V.O., who joined his regiment, the 2nd Life Guards. Before the end of 1914 thirty-three members of the staff were engaged in war work in different localities, and five more have gone this year. Considerable difficulty has been experienced in finding substitutes for those who have left the hospital for war duty, but it is satisfactory to learn that all have been triumphantly overcome, and the authorities certainly deserve high praise for the manner in which the work has been carried out, and for the fact that the benefits to out-patients have in no way decreased. One great difficulty was to find resident officers, those who were secured tending to remain a few weeks only instead of six months. Recently, in advertising for house surgeons and house physicians, it was stated that these appointments were open to women, a step without precedent in the annals of the institution.

### Movements of Students.

The circular issued by the O.C. the Medical Unit University O.T.C. advising all medical students who are yet some way from their final examination to return to their studies, has had the effect of bringing a number of men back to the school. Some have returned very reluctantly, preferring military duties to the study of medicine; one and all, however, are, we understand, working harder than ever to get through their examinations and go back to serve their country. The majority of the students are members of the O.T.C., and are actively preparing for military duties.

### Distinction for Middlesex Men.

Among Middlesex men who have gained distinction may be noted the following who have been mentioned in Sir John French's dispatches:—Major Hudleston, Major Wall, Messrs. Owen Hairsine, B. H. Wedd, of the Cancer Research Department, Lieut. Thorne, Mr. G. F. Wise-Howorth, and Mr. Cyril Helm. The latter is also the recipient of the Military Cross. Surgeon J. H. Cresswell, R.N., was awarded the bronze medal of the Royal Humane Society for the gallant rescue of an insane patient who leaped overboard. Lieut.-Colonel Walter George Pridmore has been awarded the C.M.G., and Mr. Martin H. Langford, surgeon on H.M.S. *Inflexible*, obtained the D.S.C. for his coolness in bringing up the wounded amidst suffocating fumes.

### Roll of Honour.

Six members of the school have been killed in action. Lieut. Hugh John Sladen Shields was killed while attending to a wounded man in the firing line during an attack on Reutel, eight to ten miles east of Ypres. His commanding officer, Lord Ardee, wrote of him, "The way in which he insisted on attending to wounded men under fire was the admiration of all of us." Mr. Roy Fazan was still pursuing his studies at the hospital when the war broke out. He was gazetted from the Artists' Rifle Corps to the 5th Royal Sussex, and was killed in action while in command of his company leading his men towards the German line. Mr. Keith Wood held a commission in the London Regiment, and he was killed while leading his men in a charge which resulted in the capture of some German trenches. Dr. John Henry Dauber met his death in the sunken transport *Royal Edward*, which was torpedoed. Mr. Thomas Bond Paul, lately house surgeon in the hospital, went on active service in June 1915 to the Persian Gulf, and died on September 19. Mr Charles Edgcumbe Proctor had been a resident for one year when war broke out. He obtained a commission in the Norfolk Regiment, went out to the trenches, and was shot through the head last August, having made his mark to a remarkable extent among the men of his company during his brief but brilliant military career.

* The previous article appeared on November 13, p. 143.

